# Silkworms as a factory of functional wearable energy storage fabrics

**DOI:** 10.1038/s41598-019-49193-y

**Published:** 2019-09-02

**Authors:** Basant A. Ali, Nageh K. Allam

**Affiliations:** Energy Materials Laboratory (EML), School of Sciences and Engineering, The American University in Cairo, New Cairo, 11835 Egypt

**Keywords:** Porous materials, Energy

## Abstract

Feeding *Bombyx mori* larvae with chemically-modified diets affects the structure and properties of the resulted silk. Herein, we provide a road map for the use of silkworms as a factory to produce semiconducting/metallic natural silk that can be used in many technological applications such as supercapacitor electrodes. The silkworms were fed with four different types of chemicals; carbon material (graphite), sulfide (MoS_2_), oxide (TiO_2_ nanotubes), and a mixture of reactive chemicals (KMnO_4_/MnCl_2_). All the fed materials were successfully integrated into the resulted silk. The capacitive performance of the resulted silk was evaluated as self-standing fabric electrodes as well as on glassy carbon substrates. The self-standing silk and the silk@glassy carbon substrate showed a great enhancement in the capacitive performance over that of the unmodified counterparts. The specific capacitance of the self-standing blank silk negative and positive electrodes was enhanced 4 and 5 folds at 10 mV/s, respectively upon the modification with KMnO_4_/MnCl_2_ compared to that of the plain silk electrodes.

## Introduction

Metals and semiconductors are the backbone of our modern industry. Therefore, there is a continuous need to develop new methods and technologies to produce such essential materials with the desired characteristics at low cost. Of special interest, enormous efforts have been devoted to developing flexible wearable devices. Those wearable devices are usually made of synthetic nanofibers. However, one of the cheapest and commonly used fibers is the natural silk (NS)^1,2^, which has been used, through many decades, as fabric for many applications such as biodegradable medical implants, durable protective fabrics, and eco-friendly wearable electronics^3–5^. NS consists mainly of a polymerized protein known as fibroin covered with a glue-like material named sericin^6^. It is fabricated through the organisms of silkworms from a liquid combination of polymers at room temperature, resulting in a silk that is insoluble in water^3,7^. The fibroin of the Bombyx mori larvae is a semi-crystalline biopolymer consisting of glycine, alanine and serine^8^. However, the as-produced spun silks are usually treated with additives to make them functional, which adds to the cost and requires tedious optimization. A promising approach to overcome such obstacles can occur through additives to the food of the silkworms (usually mulberry leaves)^3,8^. Feeding the worms with special chemical materials, which can be incorporated in the glands of the worms and mix with the fibroin liquid, is expected to result in a modified-silk composite that comprises the properties of both NS and the incorporated materials^6–9^. The fact that NS radiates heat more than it absorbs and self-cool, makes it a good candidate for electronic applications^10^.

Feeding Bombyx mori larvae with nanostructured materials such as CNTs^7,8^, graphene^7^, TiO_2_^9,11^ and other metal oxides^6^ have been investigated in recent reports. The feeding process proved that Bombyx mori larvae can intake nanostructured materials, which affect the crystallinity of the resulting silk. Feeding the worms with TiO_2_ was also proved to be nontoxic^11^ and even used with bacteria to enhance energy harvesting devices^12^. However, most of the previous reports were limited to the investigation of the mechanical and photonic properties of such modified silk^7,8^. Tailoring the properties of the NS to be used in electronic devices, energy generation, and energy storage devices is yet to be reported. Of special interest, flexible supercapacitors are emerging as promising platforms for energy storage^13–15^.

Herein, we demonstrate the ability to modify the structure and supercapacitive behavior of NS by feeding the Bombyx mori larvae with four different types of materials (graphite, TiO_2_ nanotubes, MoS_2_, and KMnO_4_/MnCl_2_) for use as supercapacitor electrodes. The study shows that modification of the NS enhanced its capacitive behavior, paving the way for their use in flexible supercapacitor applications.

## Materials and Methods

### Materials

The *Bombyx mori* larvae were brought from a local market in their 3^rd^ instar while the study started at the 5^th^ instar. The mulberry leaves were also brought from a local market. The graphite with particle size of 150 mesh was purchased from NICE. The TiO_2_ was prepared as reported^16^ through anodization of Ti sheet at 40 V in 0.5 M HClO_4_ electrolyte. The MoS_2_ were prepared as reported in our previous work^17^. A mixture of 1 KMnO_4_: 16 MnCl_2_ was used as a possible source for MnO_2_^18,19^. The KOH used in electrochemical measurements was purchased from AppliChem with purity 85%.

### Feeding process and silk preparation

The *B. mori* larvae were divided into 5 groups and each group has 10 larvae and were kept in a transparent dry box with good ventilation. The first group was only feeding on diet of blank mulberry leaves (S/B). While the rest 4 groups were feeding on diet of mulberry leaves previously wetted with suspensions of 0.5 wt% graphite (S/G), 0.5 wt% TiO_2_ (S/TiO_2_), 0.5 wt% MoS_2_ (S/MoS_2_) and 0.5 wt% KMnO_4_/MnCl_2_ (S/Mn), respectively. The modified diet started at the worms’ 5^th^ instar and ended by starting the spinning process. The produced cocoons were partially degummed before the characterization and the electrochemical measurements. The degumming process included drying the cocoons at 80 °C for 2 hours then the cocoons were immersed in a solution of 0.5 wt% of Na_2_CO_3_ at 100 °C for 30 minutes and this process were repeated 3 times then the cocoons were washed with distilled water for 2 minutes and repeated 3 times.

### Characterization of the produced silk

The produced silk was characterized using scanning electron microscope (SEM) (FEDEM, Zeiss SEM Ultra 60, 5 kV) the fibers were sputtered with gold at 15 A for 5 minutes before the SEM imaging. The composition of the fibroin was detected using the energy dispersive X-ray analysis (EDX) (JED 2300). The protein signals of the silk fibroin were investigated using a dispersive Raman microscope (Pro Raman-L Analyzer) with an excitation wavelength of 512 nm and Fourier transform infrared spectroscopy (FT-IR) via Perkin Elmer Spectrum One spectrophotometer using KBr pellets. The crystal structure and the change in crystal parameters were investigated using the X-ray powder diffraction (XRD) (Panalytical X’pert PRO MPD X-Diffractometer) with Cu Kα radiation (λ = 0.15418 nm, 40 kV, 30 mA). Thermogravimetric analysis (TGA) was conducted on the natural silk using the device (TGA NETZSCH STA 409 C/CD) at a heating rate of 10 °C/min and a nitrogen flaw rate of 20 ml/min.

### Electrochemical measurements

**Figure 1:**
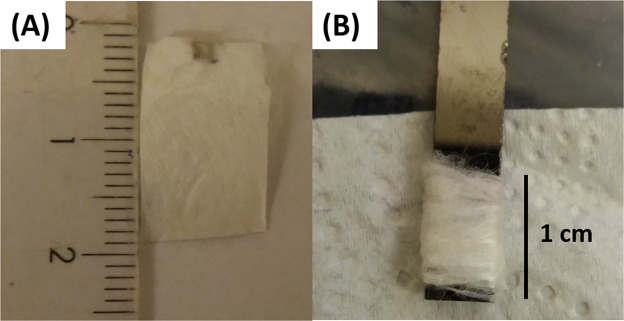
(**A)** The self-standing cocoon electrodes **(B)** The fiber@GC electrode.

The capacitive performance of the resulted silk was tested using three-electrode system in which 6 M KOH was used as the electrolyte, coiled Pt as the counter electrode, calomel electrode as the reference electrode and the silk as the working electrode. The silk working electrode was fabricated in two separate methods. To be able to test the performance of the fiber itself, the partially degummed inner layer of the cocoons was cut into a square of 2 × 1 cm^2^ area as presented in Fig. 1(A) and a drop of Ag paste was used as a current collector on the point of attachment to the crocodile of the potentiostat. Half the piece of fiber was immersed in the electrolyte and half the weight of the fiber was taken as the weight of the active material in the electrode. Since the electrolyte upwards in the fiber besides the fiber piece was not homogeneous so another method was used to assure the results. The strands of fiber were weighted and coiled over a length of 1 cm of a glassy carbon (GC) rod as presented in Fig. 1(B). The part covered with the fiber was immersed completely in the electrolyte and used as the working electrode. The electrochemical measurements were performed using BioLogic SP-300 potentiostat and included measuring cyclic voltammetry (CV) in potential windows (0.0 to 0.4) and (−0.4 to 0.0) in order to identify the performance of the active materials as positive and negative electrodes respectively. The cyclic voltammetry was measured at different scan rates (10, 50, 100 and 500 mV/s). The capacitance was measured form the cyclic voltammogram using Eq. 1. The capacitive performance can also be calculated using Eq. 2 from the charge/discharge measurement. The galvanostatic charge/discharge measurement (GCD) was performed at different applied currents (0.1 to 0.5 A/g). The stability of the silk fibers was measured up to 1000 cycle at applied current of 0.1 A/g. The electrochemical impedance spectroscopy (EIS) of the system was measured at frequency range between 1 MHz to 100 mHz. The measurements were repeated twice on two different samples from each type of fibroin

1 $${{\rm{C}}}_{{\rm{s}}}=\frac{\int {\rm{IdV}}}{{\rm{vm}}\,{\rm{\Delta }}{\rm{V}}}$$

C_s_ is the specific capacitance, I is the response current density, ν is the potential scan rate, ΔV is the potential window, and m is the mass of electrode material.

2 $${{\rm{C}}}_{{\rm{sp}}}=\frac{{\rm{I}}\,{\rm{dt}}}{{\rm{m}}\,{\rm{dV}}}$$

dt is the discharging time (s), I is the discharging current (A), m is the mass of the active material (g) within the electrode, and dV is the discharging potential range (V).

## Results and Discussion

### Effect of the feeding process

All the studied silkworms started the feeding on their 5^th^ instar and they did not reject the food. It was observed that the larvae fed with MoS_2_ were eating more than usual while the ones fed with KMnO_4_/MnCl_2_ were eating in a lower rate than usual. The larvae fed with graphite and TiO_2_ did not show any unusual behavior in the feeding process. While the Cocoons of the blank fed larvae were of homogeneous size and white color, the chemically-modified ones showed a non-homogenous size and off-white color. After degumming, all the fabricated fibers were of a clear white color. The resulted silk was given the names S/B, S/G, S/TiO_2_, S/MoS_2_ and S/Mn for the blank silk, the graphite modified silk, the TiO_2_ modified silk, the MoS_2_ modified silk and the KMnO_4_/MnCl_2_, respectively.

### Structure of the resulted silk

**Figure 2:**
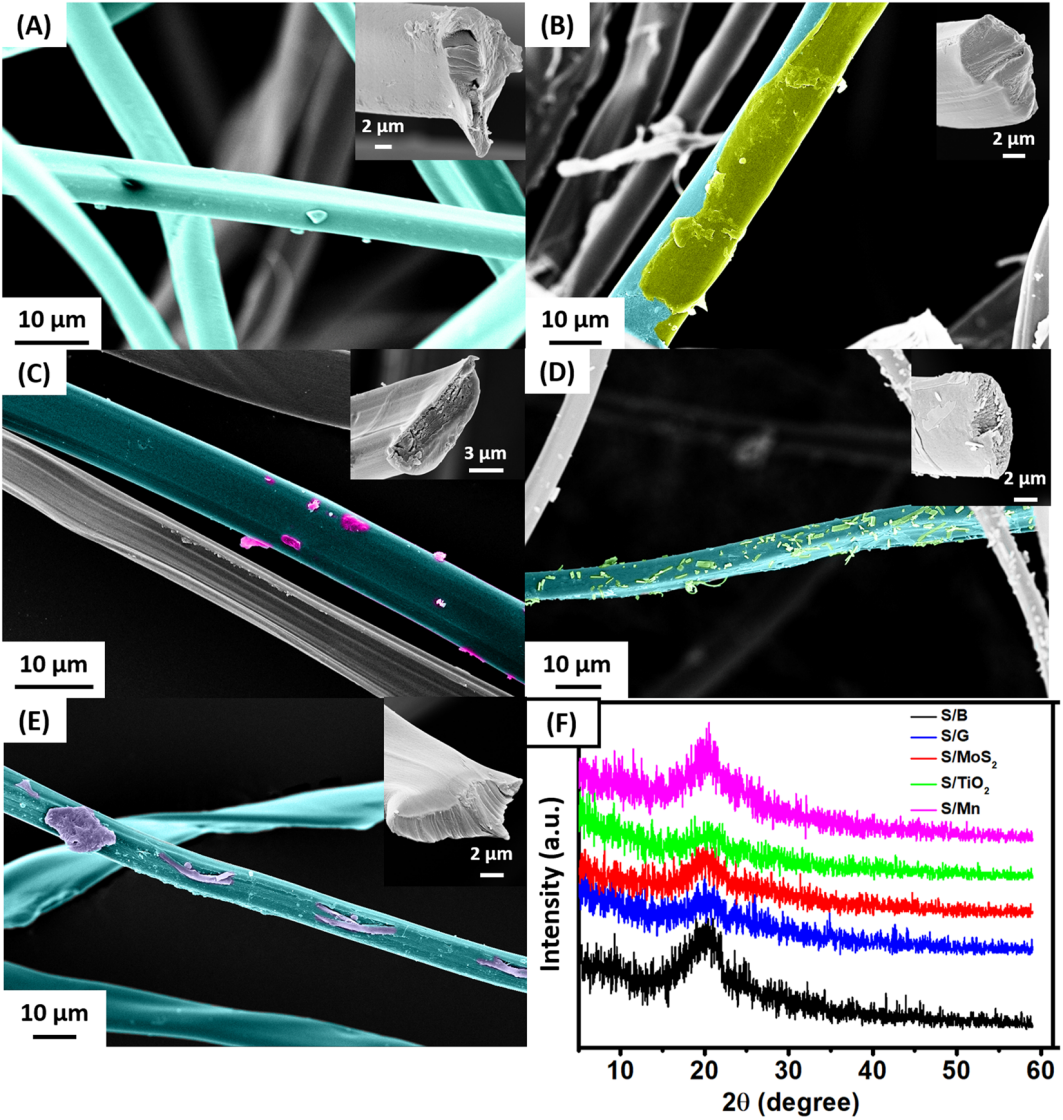
Morphological and structural analysis of the silk: **(A–E)** FESEM images of the fabricated fibers (inset: cross section in the fiber) “pseudo-color is used for clarity” **(A)** S/B, **(B)** S/G, **(C)** S/MoS_2_, **(D)** S/TiO_2_, **(E)** S/Mn, and **(F)** the corresponding XRD patterns.

The morphology of the silk fibers was investigated using FESEM imaging as shown in Fig. 2. Note that the thickness of the fabricated fibers is independent of the type of the chemical additive, having diameters ranging from 9 to 16 µm, in agreement with previous reports^8,20^. The fed materials appeared as debris on the surface of the fibers and/or within their internal fibroins. While the S/B fibers showed a trigonal shaped cross-section as presented in the inset of Fig. 2, the S/G and S/TiO_2_ showed an oval-shaped cross-section with the additives clearly appearing on the surface of the fibers. However, the S/MoS_2_ and S/Mn showed a flattened oval cross-section and the fibers were more flat than usual, which may suggest that the additives (MoS_2_, Mn) were interfered with the fiber materials and reconstructed its protein structure^8^. The elemental composition of the fibers was studied using the EDS technique and the results are presented in Table 1. The resulted composition showed that the added material did not exceed 0.03 at% of the total atoms in the fiber, which is an accepted ratio due to the low concentration (0.5 wt% suspension) used in the diet. The S/B and S/G did not vary greatly due to the fact that graphite is only made of carbon atoms. However, the S/MoS_2_ analysis showed 0.03 at% of Mo and 0.05 atom% of S. the S/TiO_2_ showed a Ti composition of 0.03 at% and the S/Mn showed 0.02 at% of Mn and 0.01 at% of K “from the added KMnO_4_”, with no signal for Cl atoms at different positions of the S/Mn fibers indicating that Cl_2_ gas may have evaporated from the reaction medium during the formation of MnO_2_^19^. Although the EDS analysis showed a minor ratio of the added materials, the SEM images showed a major effect on the morphology of the resulted fiber. The investigation of the crystal structure of the silk was performed using XRD as presented in Fig. 2(F). The XRD patterns show that all the resulted silk has a mesophase behavior with a broad peak around 20.0°, which can be attributed to the β-sheet of silk II structure^21–25^. The mesophase structure of the silk is believed to facilitate the diffusion of ions to the internal parts of the silk fibers.

**Table 1:** EDS analysis of the spun silk.

Material	C (atom%)	N (atom%)	O (atom%)	Mo (atom%)	S (atom%)	Ti (Atom%)	Mn (atom%)	K (atom%)	Cl (atom%)
S/B	81.94	10.31	7.75	N/A	N/A	N/A	N/A	N/A	N/A
S/G	82.24	9.84	7.92	N/A	N/A	N/A	N/A	N/A	N/A
S/MoS_2_	81.9	10.13	7.89	0.03	0.05	N/A	N/A	N/A	N/A
S/TiO_2_	85.96	6.86	7.15	N/A	N/A	0.03	N/A	N/A	N/A
S/Mn	81.36	9.55	9.06	N/A	N/A	N/A	0.02	0.01	N/A

**Figure 3:**
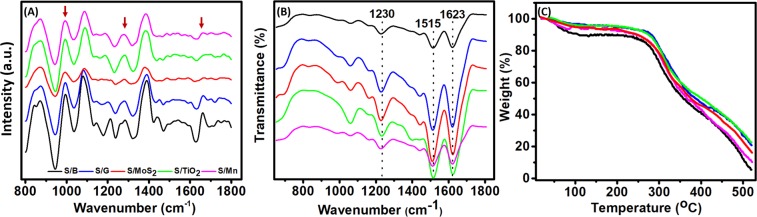
The characterized peaks of the spun silk fibers **(A)** Raman spectroscopy, **(B)** FT-IR, and **(C)** TG analysis for the silk fibers.

As the Raman spectroscopy has been used as a good tool to investigate the deformation of polymers backbone structure^26^, the Raman spectra of the fabricated silk were recorded as shown in Fig. 3(A). All fibers showed the same peak position with different intensities, indicating more or less a similar internal structure. The Raman active peaks of the studied fibers are in the range between 800 to 1800 cm^−1^, in a good agreement with literature^4,26^. The observed Raman peaks of the B. Mori silk appeared at 1085, 1232 and 1669 cm^−1^ as indicated by red arrows in Fig. 3(A). The FTIR spectra in Fig. 3(B) showed the typical peaks at 1623, 1515 and 1230 cm^−1^ characteristic of the silk fibers but with different intensities for different samples^6,8,9^. The peak at 1623 cm^−1^ indicated the presence of amide I structure, which can be ascribed to the vibration of the C=O bond due to the co-formation of the α-helix and random coiled structures. The peak at 1515 cm^−1^ indicated the presence of amide II structure, which can be related to the deformation of the N-H bond in the β sheet structure. The Peak at 1623 cm^−1^ indicated the presence of amide III structure and the peak is due to the vibration of the O-C-O bonds and the N-H bond. The positions of the peaks did not change with the chemical additives, which confirm that the chemical additives did not change the original backbone of the silk fibroins and hence the mechanical properties. CasaXPS software^27^ was used to deconvolute the peaks of the FTIR, which indicated that the percentage of both α-helix and β sheet structure are almost equal in all samples and the presence of the β-sheet structure was found to be more pronounced in the S/TiO_2_, the blank silk, and the S/G samples more than in the S/Mn, and the S/MoS_2_ samples, the deconvoluted data are presented in Table S1. Thermogravimetric analysis was performed to indicate the thermal stability of the resulted silk fibers. Figure 3(C) shows that all the silk fibers were stable up to 200 °C then the blank silk started to decompose at ~250 °C. The modified silk showed enhanced thermal stability. At 500 °C, the remaining weight of the silk was 28.18, 26.7, 21.67, 16.13 and 12.27% for S/TiO_2_, S/G, S/MoS_2_, S/Mn, and S/B, respectively.

### Electrochemical performance of the natural silk

**Figure 4:**
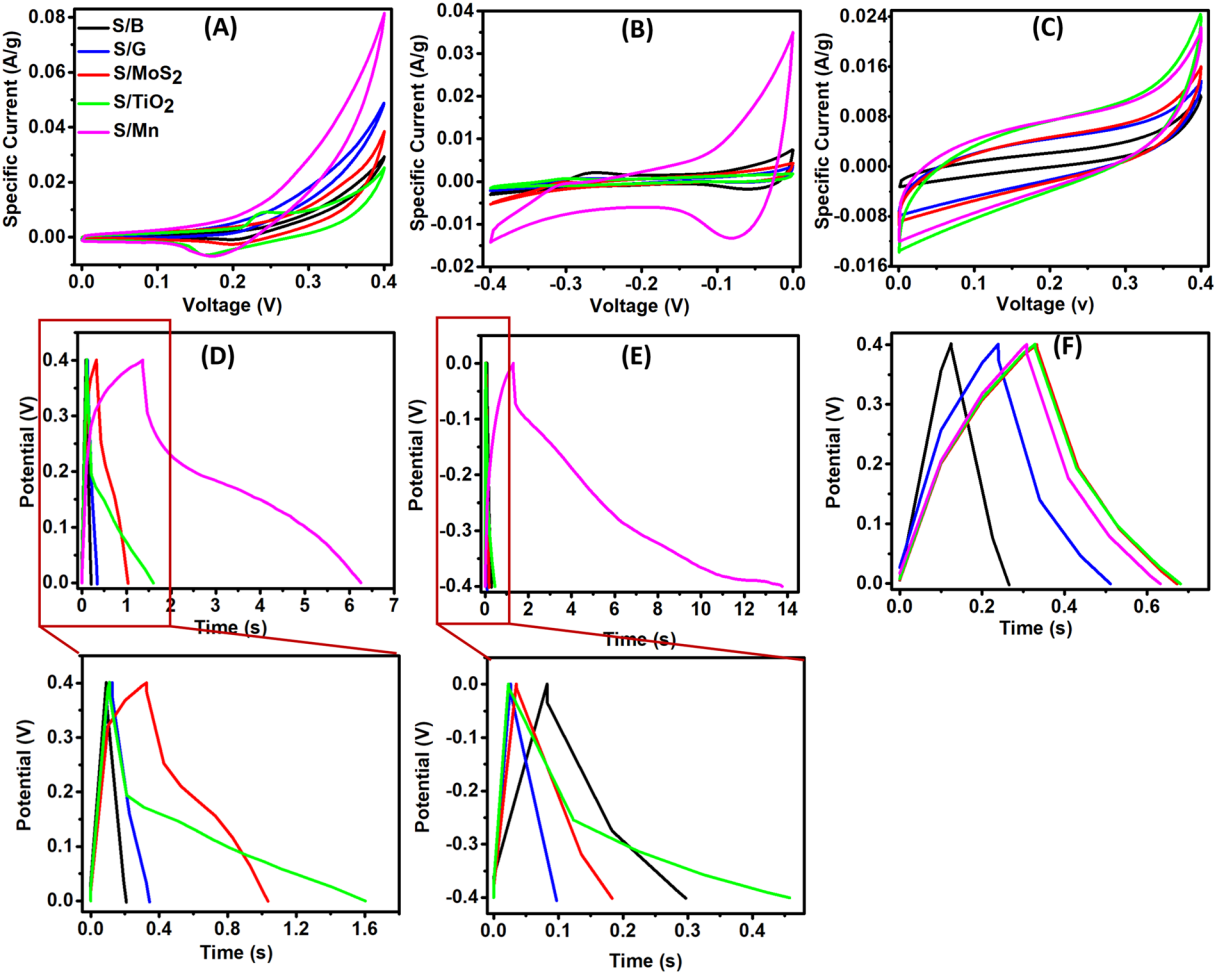
Electrochemical performance of the silk fibers: **(A)** CVs of the studied self-standing silk fiber at 10 mV/s in positive potential window (inset: legend of **(A–F)**), **(B)** CVs of the studied self-standing silk fiber at 10 mV/s in negative potential window, **(C)** CVs of the studied silk @ GC at 10 mV/s in positive potential window, **(D)** GCDs of the studied self-standing fibers at 0.1 A/g in positive potential window (inset: enlarged figure), **(E)** GCDs of the studied self-standing fibers at 0.1 A/g in negative potential window (inset: enlarged figure), **(F)** GCDs of the studied silk @ GC at 0.1 A/g in negative potential window.

To test the capacitive performance of the natural silk, the self-standing silk was tested once as a positive electrode and once as a negative electrode in a 3-electrode system with 6 M KOH as the electrolyte. Although 6 M KOH is a high concentration electrolyte, it is commonly used with the carbon-based materials in supercapacitor applications^28–33^. Usually, the carbon materials show a typical rectangular cyclic voltammogram (CV) reflecting the electrical double layer behavior (EDL)^34–36^. However, the CVs of the positive and negative silk electrodes in Fig. 4(A,B) did not show an EDL behavior indicating diffusion processes for the ions in the polymeric structure of the silk^34^. It is expected that the OH- ion from the KOH reacted with the organic polymer of the silk fibers resulting in a diffusion and pseudocapacitive behavior to the silk electrodes. The ions from the KOH can react with MoS_2_, TiO_2_, and MnO_2_ to give MoSSOH^37^, TiOOK^38^ and MnOOK^39^, respectively. The CVs of the positive silk electrodes at a scan rate of 10 mV/s (Fig. 4(A)) show that the redox peaks are more visible in the S/TiO_2_ while the other additives did not affect the shape of the CV of the S/B. This can be ascribed to the accumulation of TiO_2_ on the surface of the silk fibers while other additives affected the morphological shape of the silk fibers and did not accumulate with high amount on the surface of the fibers. At a scan rate of 10 mV/s and at a positive potential window, the specific capacitance of the S/Mn showed the highest specific capacitance of 778.975 mF/g while the S/TiO_2_, S/MoS_2_, S/G and S/B showed 577.925, 419.767, 247.822, and 157.291 mF/g, respectively. This shows that all the additives dramatically increased the specific capacitance values of silk electrodes. The CVs of the negative silk electrodes at a scan rate of 10 mV/s (Fig. 4(B)) show clearer redox peaks than the positive electrodes. The specific capacitance of the negative electrodes calculated at a scan rate of 10 mV/s was 1122.832, 263.047, 131.794, 112.141, and 109.403 mF/g for S/Mn, S/B, S/MoS_2_, S/G and S/TiO_2_, respectively. To make a deeper study with accurate weight of the active material, the strands of the silk fibers were coiled over a glassy carbon (GC) electrode and measured as a positive electrode. The calculated specific capacitance of silk fibers @ GC at 10 mV/s (Fig. 4(C)) showed a capacitance of 610.911, 604.701, 569.047, 556.923, and 206.650 mF/g for S/MoS_2_, S/TiO_2_, S/Mn, S/G and S/B, respectively. The contribution of the GC current collector affected the shape of the CVs and shifted them to the EDL rectangular shape. Also, the GC affected the values of the specific capacitance and the order of the materials in their capacitance values. Therefore, the current collector affects greatly the overall performance of the material and we will focus herein on the self-standing fibers as they are more reliable for the study. As one of the most important metrics of supercapacitors is their ability to store and release charges, the time of the charge and discharge was also studied for the silk fibers. Figure 4(D,E) shows the galvanic charge/discharge (GCD) curves of the self-standing silk fibers at a current density of 0.1 A/g. The GCD curves show a pseudocapacitive behavior^34^. For the positive electrodes, the specific capacitance calculated from the GCD at 0.1 A/g showed the same trend as that calculated from the CVs at 10 mV/s. The specific capacitance values of the positive electrodes calculated at 0.1 A/g were 1222.2, 373.25, 177.2, 54.6, and 29.25 mF/g for S/Mn, S/TiO_2_, S/MoS_2_, S/G and S/B, respectively. However, for the negative electrodes, the specific capacitance values calculated at 0.1 A/g were 3114, 108.7, 53.6, 37.05, and 17.8 mF/g for S/Mn, S/TiO_2_, S/B, S/MoS_2_ and S/G, respectively. The GCD curves of the silk @ GC positive electrodes at 0.1 A/g are presented in Fig. 4(F). The specific capacitance of the positive silk @ GC calculated at 0.1 A/g were 88.3, 85.1, 81.05, 68.1, and 35 mF/g for S/TiO_2_, S/MoS_2_, S/Mn, S/G and S/B, respectively. As for the CV results of the silk @ GC, the trend is different, and the effect of the current collector is shifting the shape of the GCD curves to the ideal shape of the EDL capacitor materials. However, the specific capacitance values of the silk with additives are still much higher than this of the blank silk. The CV and GCD results showed that the blank silk (S/B) behaved better as a negative electrode than as a positive electrode and so did the addition of Mn ions (S/Mn) and usually MnO_2_ acts as a better capacitive material when used as a negative electrode^19^. However, the S/G, S/MoS_2_ and S/TiO_2_ enhanced the performance of the silk as a positive electrode than as a negative electrode. Although the amounts of the additives were relatively low, their effect can be attributed to both the nature of the materials and their effect on the morphology of the silk fiber, which controls the diffusion of ions into the silk fibers.

**Figure 5:**
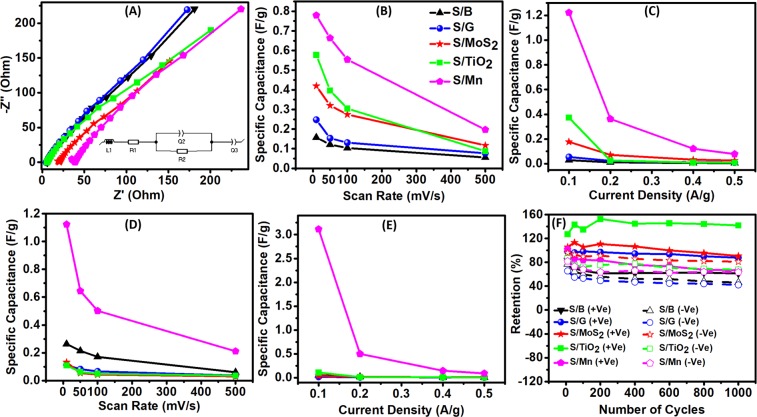
Electrochemical stability of the silk fibers **(A)** Nyquist plots of the studied silk @ GC in the range 1 MHz to 100 mHz (inset: fitting circuit and fitting curve). **(B)** Change of specific capacitance with scan rate (10, 50, 100, and 500 mV/s) for the self-standing fiber in positive potential window (inset: legend for **(A–E)**), **(C)** Change of specific capacitance with current density (0.1, 0.2, 0.4 1nd 0.5 A/g) for the self-standing fiber in positive potential window, **(D)** Change of specific capacitance with scan rate (10, 50, 100, and 500 mV/s) for the self-standing fiber in negative potential window, **(E)** Change of specific capacitance with current density (0.1, 0.2, 0.4 1nd 0.5 A/g) for the self-standing fiber in negative potential window, **(F)** Retention of the studied self-standing fiber in both positive and negative potential window.

The conductivity is one of the main factors that affects the overall performance of a supercapacitor electrode. Figure 5(A) shows the Nyquist plots of 10 mg of silk fibers coiled over the same area of a glassy carbon electrode. The resulted curves were fitted to the inset circuit in Fig. 5(A), with R1 representing the electrolyte resistance and R2 representing the charge transfer resistance of the material. As the obtained circle is a depressed semicircle not a perfect semicircle, Q was used in the fitting instead of C and L is used to represent the inductance related to the electrical connections, Z’ and Z” represent the real part and the imaginary part of the impedance, respectively. This circuit showed a perfect match with all the Nyquist plots as presented in Fig. S1. The R2 values of the silk fibers were 157.7, 115.7, 104.9, 92.54, and 39.72 Ω for S/B, S/G, S/TiO_2_, S/MoS_2_ and S/Mn, respectively. Those R2 values show that the additives greatly enhanced the conductivity of the silk fibers and hence enhanced their specific capacitance. The supercapacitors should be able to work under different conditions of scan rates and current densities. The value of the specific capacitance of self-standing silk positive electrodes versus the scan rate is presented in Fig. 5(B). Note that the specific capacitance values have the same trend except at 500 mV/s. At 500 mV/s, the specific capacitance values are 196.991, 117.22, 87.491, 76.976, and 55.531 mF/g for S/Mn, S/MoS_2_, S/TiO_2_, S/G, S/B, respectively. On the other hand, from the GCD calculations of the positive self-standing silk electrodes (Fig. 5(C)), the trends differed over the high current density. It showed the values of 78.25, 26.5, 9.5, 8.75, and 5.5 mF/g at 0.5 A/g for S/Mn, S/MoS_2_, S/G, S/TiO_2_, and S/B, respectively. For the negative self-standing silk electrodes, the change of specific capacitance with scan rate is presented in Fig. 5(D). The values of the S/Mn and S/B were always much higher than those of the S/G, Si/MoS_2_, and Si/TiO_2_. At a scan rate of 500 mV/s, the specific capacitance values of the negative self-standing electrodes were 211.009, 60.195, 36.27, 35.729, and 30.272 mF/g for S/Mn, S/B, S/G, S/TiO_2_, and S/MoS_2_, respectively. The trend of the specific capacitance at different current densities is presented in Fig. 5(E) and enlarged in Fig. S2. At a current density of 0.5 A/g, the specific capacitance values of the negative self-standing electrodes were 90.5, 12, 7.75, 6.8, and 5.25 mF/g for S/Mn, S/MoS_2_, S/G, S/TiO_2_, and S/B, respectively. Although the S/TiO_2_, S/MoS_2_, and S/G specific capacitance values as negative electrode (from GCD) are higher than that of the S/B but it is lower than their positive electrode values (from GCD). Thus, it is believed that S/TiO_2_, S/MoS_2_, and S/G act better as positive electrodes than as negative electrodes. Despite the different trends over the different scan rates and current densities, the performance of all silk with additives was better as positive electrodes than the blank silk and the S/Mn was always better as a negative electrode. One of the performance metrics of the supercapacitor materials is their stability upon cycling. Figure 5(F) shows the retention percentage of the self-standing silk as positive and negative electrodes over 1000 cycles. The retention fluctuates at the first 200 cycles and reaches a relative stability after 600 cycles. The positive electrodes showed retention of 141.88, 90.59, 87.7, 66.63, and 61.3% for S/TiO_2_, S/MoS_2_, S/G, S/Mn, and S/B, respectively after 1000 cycles. The negative electrodes showed retention of 80.99, 67.6, 63.45, 46.06, and 42.13% for S/MoS_2_, S/TiO_2_, S/Mn, S/B, and S/G, respectively. From the retention results we conclude that the silk fiber has a better retention as a positive electrode in general and that the additives enhanced the retention and cyclability of the electrodes. the above 100% retention values are attributed to the further diffusion of ions into the material and enhancement of reaction over time^17,40^. Noteworthy to mention that the specific capacitance values in mF are acceptable for self-standing carbon-based materials with no high conductive current collectors^41–43^.

## Conclusions

We demonstrate the ability to fabricate functionalized natural silk fibers by feeding the silkworms with the material of interest. Specifically, this work highlights the possibility of using natural silk fibers as supercapacitor electrodes upon feeding the worms with high capacitive materials such as graphite, MoS_2_, TiO_2_, and KMnO_4_/MnCl_2_. The study showed that the fed material did not greatly affect the crystallinity of the silk fibroin and all the added materials enhanced the capacitance performance and the thermal stability of the silk fibers. It was observed that both S/B and S/Mn contained more β-sheet silk, have close thermal stability, and both acted better as negative electrodes. The study proved that natural silk can be tuned for use in energy storage devices.

## Supplementary information

Supporting information
