# Supplementary material for: Silkworms as a factory of functional wearable energy storage fabrics
Source: Sci Rep. 2019 Sep 2;9:12649. doi: 10.1038/s41598-019-49193-y (PMC6718607; doi:10.1038/s41598-019-49193-y)
Supplement: Supplementary file 1 — Supporting information [file 41598_2019_49193_MOESM1_ESM.pdf]

# Supporting Information

# Silkworms as a factory of functional wearable energy storage fabrics

**Basant A. Ali[1] & Nageh K. Allam[1,]***

[1]Energy Materials Laboratory (EML), School of Sciences and Engineering, The American University in Cairo, New Cairo 11835, Egypt.
* Correspondence and requests for materials should be addressed to N.K.A. (nageh.allam@aucegypt.edu).

**Table S1** Deconvoluted data of the FTIR spectra.

| Material | Area under the peak of ~1515 $cm^{-1}$ (β sheet) | Area under the peak of ~1623 $cm^{-1}$ (α-helix) |
|---|---|---|
| **S/B** | 9.37 | 9.65 |
| **S/G** | 10.03 | 11.49 |
| **S/$MoS_2$** | 6.40 | 7.26 |
| **S/$TiO_2$** | 12.15 | 13.47 |
| **S/Mn** | 4.24 | 4.83 |

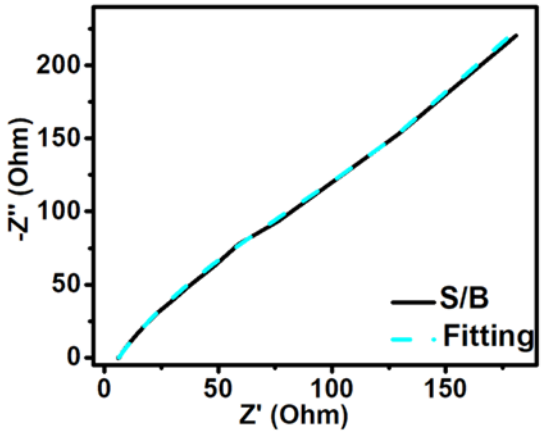


**Fig. S1** Fitting of the impedance spectroscopy of S/B fibers.

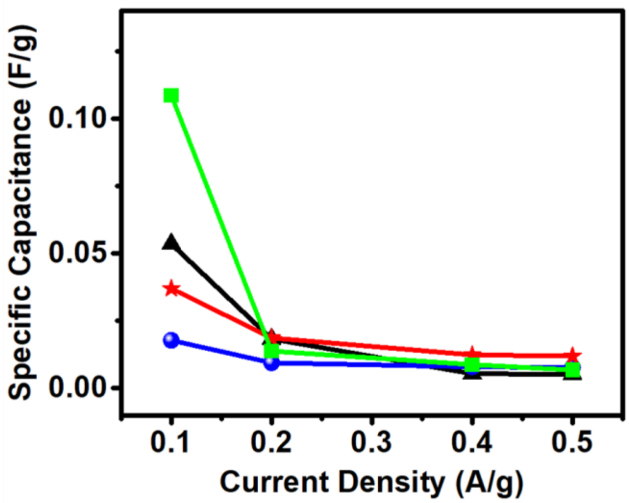


**Fig. S2** Enlargement of the change of specific capacitance with current density (0.1, 0.2, 0.4 and 0.5 A/g) for the self-standing fiber in negative potential window
